# Balancing Cost and Precision: An Experimental Evaluation of Sensors for Monitoring in Electrical Generation Systems

**DOI:** 10.3390/s25227052

**Published:** 2025-11-18

**Authors:** Janeth Alcalá, J. Antonio Juárez, Víctor Cárdenas, Saida Charre-Ibarra, Juan González-Rivera, Jorge Gudiño-Lau

**Affiliations:** 1 Faculty of Electromechanical Engineering, University of Colima, Manzanillo 28860, Colima, Méxicoscharre@ucol.mx (S.C.-I.); jglau@ucol.mx (J.G.-L.); 2 Engineering Department, Autonomous University of San Luis Potosí, San Luis Potosí 78290, San Luis Potosí, México; vcardena@uaslp.mx (V.C.); juan.gonzalez@uaslp.mx (J.G.-R.)

**Keywords:** current sensor, voltage sensor, energy management, scalable energy systems, photovoltaic systems

## Abstract

**Highlights:**

**What are the main findings?**

- The comparison demonstrates that low-cost devices are suitable only for general trend visualization, while high-precision sensors are required for accurate voltage and current measurements.
- Low-cost sensors exhibit deviations greater than 5%, whereas high-precision sensors maintain errors below 1% when monitoring generation systems.

**What are the implications of the main findings?**

- Accurate monitoring of generation systems depends on the use of high-precision sensors, particularly for performance assessment and operational decision-making.
- As PV adoption continues to grow, reliable sensing becomes critical for scalable, long-term monitoring solutions.

**Abstract:**

The growing adoption of renewable energy conversion systems and smart infrastructures has increased the demand for accurate monitoring solutions to ensure system performance and reliability, as well as seamless integration with cloud-based platforms. Voltage and current sensing are central to this task; however, sensor selection often involves a trade-off between cost and measurement precision. Rather than comparing technologies as equivalent options, this study investigates the practical impact of using low-cost versus high-precision sensors in electrical power generation monitoring. The evaluation includes representative low-cost sensors and high-precision alternatives based on instrumentation amplifiers and a closed-loop Hall-effect transducer. All sensors were characterized under controlled laboratory conditions and analyzed using statistical indicators, including MAE, RMSE, MAPE, and R^2^. Results show that high-precision sensors achieved R^2^ > 0.97 and MAPE < 4%, whereas low-cost sensors showed R^2^ as low as 0.73 and errors exceeding 10% under dynamic irradiance conditions. Low-cost sensors present deviations of 5–8% in RMS measurement, while high-precision sensors maintain error below 1%.

## 1. Introduction

In recent years, the rapid growth of distributed generation systems has gained significant attention due to their potential to improve energy efficiency, reduce operational costs, and mitigate environmental impact. Among these systems, photovoltaic (PV) networks have emerged as a key technology for decentralized power generation. However, ensuring the reliability and efficiency of these systems requires advanced monitoring solutions capable of capturing accurate electrical measurements.

Voltage and current sensors are fundamental components of such monitoring frameworks, as they enable precise measurements for energy management, control strategies, and fault detection. Despite the wide availability of commercial sensors, selecting devices that simultaneously deliver high accuracy, reliability, and cost-effectiveness remains a challenge.

The study presented in [1] offers a comprehensive analysis of how different IoT components, including sensors, can significantly enhance the operational efficiency and predictive capabilities of systems such as PV networks and microgrids. The integration of such technologies into smart energy management systems not only enables real-time monitoring but also supports the development of advanced control strategies for microgrids and large-scale energy systems.

The detailed analysis of low-cost current sensors for building energy management in [2] underscores the necessity for accurate and reliable sensors to ensure effective energy monitoring systems. Their findings demonstrate that while the PZEM-004T sensor offers high accuracy, the ACS712 sensor provides a more cost-effective solution with moderate accuracy, making it suitable for cost-sensitive applications.

Several studies have investigated monitoring systems within the context of Home Energy Management Systems (HEMS) [3,4,5,6,7]. However, these works do not specifically address the challenges and requirements associated with monitoring in distributed generation environments, as their primary focus lies elsewhere. Consequently, they often assume that the proposed solutions are scalable to other contexts, without directly assessing the accuracy and reliability of the sensors under the operational conditions characteristic of distributed energy systems.

In [8], the authors use a low-cost energy sensor (PZEM-004T) alongside ESP32 microcontrollers, to demonstrate the potential of integrating advanced regression models and kernel density estimation for predictive accuracy and real-time monitoring. Their system exemplifies how robust sensor integration and predictive analytics can significantly enhance operational efficiency in energy management. Unlike predictive approaches such as [8], which compensate sensor inaccuracy through regression models and statistical post-processing, the present study isolates the physical performance of the sensors themselves. This distinction is relevant because software-based correction depends on prior calibration, training datasets, and operating conditions, whereas measurement uncertainty is intrinsic to the sensing hardware.

Additionally, studies such as [7,9,10,11,12] propose using low-cost sensors, including the SCT013 and ZMPT101B, for energy monitoring applications. In [10], these sensors are integrated into an IoT-based architecture for real-time energy monitoring, employing advanced data analysis techniques to enhance the accuracy of power consumption forecasting. Furthermore, works such as [7,9,10] highlight the versatility and operational reliability of these sensors across diverse scenarios, encompassing industrial energy monitoring within Industry 4.0 frameworks, as well as residential and academic settings.

By integrating SCT013 and ZMPT101B sensors into IoT and edge-computing platforms, recent studies highlight their potential to provide real-time current and voltage measurements with sufficient precision to support energy forecasting, enhance operational efficiency, and enable scalable monitoring architectures in both industrial and residential environments. Nevertheless, despite demonstrating satisfactory performance, these studies do not present a quantitative assessment of the sensors’ measurement accuracy.

In [13], the authors built an energy monitoring system for PV systems using different low-cost sensors, an Arduino UNO board for data processing, and an ESP8266 module for Wi-Fi communication. However, the study does not include a detailed analysis of sensor accuracy nor an evaluation of the measurement reliability, leaving open questions regarding the suitability of such devices in performance-critical systems. Likewise, in [14], the authors present a battery-monitoring system for PV installations using LoRa communication and low-cost sensors, but again no statistical assessment of measurement error is reported.

Although the performance difference between low-cost and high-precision sensors is generally acknowledged, recent literature shows that low-cost sensing devices are routinely incorporated into PV monitoring platforms, energy dashboards, and IoT-based data acquisition systems without formal validation against reference instruments or real operating conditions. As a result, their measurement error may propagate into higher-level processes such as fault detection, power forecasting, inverter control, grid reporting, performance-ratio calculation, and LCOE (Levelized Cost of Energy) estimation. Nevertheless, most reported studies assume that the proposed sensing systems can be directly applied to photovoltaic measurement applications, without quantitatively evaluating their measurement accuracy or linearity. To the best authors’ knowledge, no controlled and statistically validated comparison between low-cost and high-precision sensors under both laboratory and field conditions has been reported. Providing such quantitative evidence is essential, as it enables informed sensor selection and prevents bias in monitoring, diagnosis, and system-level performance evaluation.

In response to these limitations, this research evaluates and compares the performance of representative low-cost sensors and high-precision alternatives. The concern is not limited to the fact that low-cost sensors have greater uncertainty, but to the fact that they are frequently integrated into monitoring platforms without prior validation of their measurement accuracy. When this assumption goes unverified, sensor errors may propagate into performance metrics, diagnostic algorithms, and research datasets, leading to biased conclusions and unreliable decision-making in photovoltaic systems.

The evaluation is conducted through controlled laboratory characterization and validated in field PV operation under naturally fluctuating irradiance and weather conditions. Statistical metrics including mean absolute error (MAE), root mean square error (RMSE), mean absolute percentage error (MAPE), and the coefficient of determination (R^2^) are used to quantify performance differences. The results provide quantitative evidence of the limitations of low-cost sensors and identify the conditions under which high-precision sensors are required for reliable monitoring in electrical power generation systems. A custom ESP32-based monitoring system was developed to acquire, process, and transmit real-time data to cloud platforms, enabling a complete evaluation of each sensor’s performance in practical operating scenarios. The study characterizes the accuracy and linearity of both low-cost and high-precision sensors under controlled laboratory conditions and validates their performance in a photovoltaic (PV) system operating under real-world weather conditions in field environments. The results provide useful criteria for selecting sensors in electrical power generation monitoring systems, emphasizing the trade-offs between cost and accuracy and the relevance of high-precision devices when reliable measurements are required. The main contributions of this work are threefold: (1) a controlled experimental comparison of low-cost and high-precision sensors using standardized statistical metrics, (2) validation of sensor performance under field operating conditions in a photovoltaic (PV) system, extending beyond laboratory simulation, and (3) the development of an open-source monitoring platform that enables reproducible benchmarking of sensor accuracy and integration workflows.

The remainder of this paper is as follows: Section 2 describes the methodology, including sensor comparison and selection, the proposed monitoring board design, sensor characterization with simulation and experimental validation, and the signal processing algorithm. Section 3 presents the results of the integration of these sensors with the PV system in an experimental setup under different weather conditions. Section 4 discusses the main findings, and Section 5 concludes with contributions and outlines future research directions.

## 2. Experimental Setup and Sensor Characterization Methodology

This study aims to evaluate the performance of low-cost voltage and current sensors to determine their suitability for monitoring applications in electrical power systems. Rather than conducting a direct benchmarking exercise, the objective is to identify the operational constraints, accuracy limitations, and signal-conditioning requirements that emerge when these sensors are deployed in real-world measurement scenarios, including both laboratory and field environments.

The methodology comprises four main stages: (i) selection of commercially available low-cost sensors, (ii) design and implementation of a modular monitoring board, (iii) development of the data acquisition and signal-processing pipeline, and (iv) characterization of sensor performance under both controlled laboratory conditions and field operating environments.

### 2.1. Sensor Comparison and Selection

Based on the literature review, two representative low-cost sensors were selected: the ZMPT101B for voltage measurement and the SCT-013 for current sensing. These devices are widely used in educational platforms and IoT-based prototypes due to their affordability and simplicity of integration. However, their single-ended analog output, lack of factory calibration, and limited electromagnetic immunity introduce non-linearity and drift when deployed under field operating conditions.

For high-precision alternatives, isolated Hall-effect–based sensors were considered due to their inherent galvanic isolation, differential output architecture, low intrinsic noise, and traceable calibration procedures [15]. These features enable superior linearity and temperature stability compared to low-cost transducers, whose accuracy depends heavily on external filtering and software correction. In addition, isolated Hall-effect sensors maintain accuracy over a wide dynamic range and are less susceptible to common-mode interference, making them suitable for power electronics and renewable-energy metrology.

Accordingly, an isolated voltage sensing stage was implemented using the HCPL-7800 optocoupler and the OP127GSZ low-drift operational amplifier, while current measurement was performed using the HXS20-NP Hall-effect sensor. The HXS20-NP is designed for precision measurement applications and provides high linearity, galvanic isolation, low insertion loss, and wide bandwidth—characteristics that are essential when accurate monitoring is required in photovoltaic systems and distributed power generation environments.

### 2.2. Monitoring Board Design

Table 1 presents a comparison of the selected sensors, highlighting their key specifications and performance characteristics. This comparative analysis informed the design of signal conditioning and adaptation circuits to ensure compatibility with the ESP32 microcontroller.

The ESP32 microcontroller provides an extensive set of features, including integrated Wi-Fi and Bluetooth connectivity, low power consumption, multiple 12-bit ADC channels, and a dual-core processor operating at up to 240 MHz. Owing to its low cost, versatility, and processing capability, it is particularly suitable for real-time energy monitoring applications.

#### 2.2.1. Low-Cost Sensor Configuration

**Figure 1:**
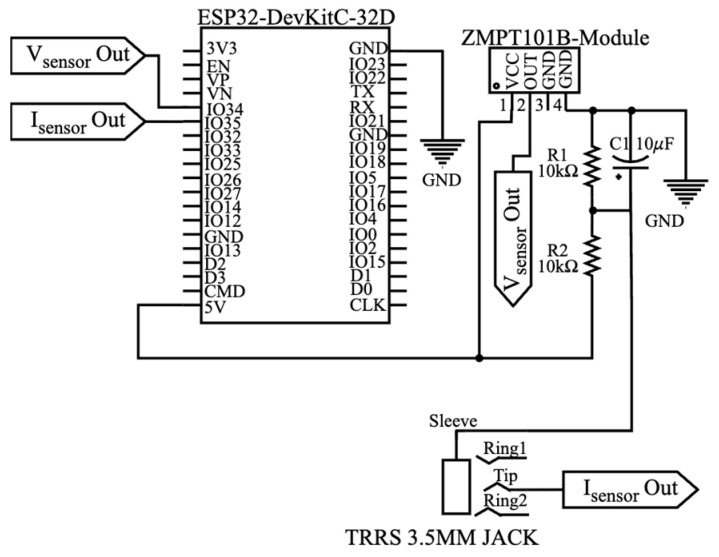
Monitoring board design with low-cost sensors.

A signal conditioning stage was implemented to ensure proper interfacing between the sensors and the ESP32. The wiring diagram for the monitoring board is shown in Figure 1. The SCT-013 current sensor required a voltage divider with a DC offset to center its output signal within the ESP32’s input range. In contrast, the ZMPT101B voltage sensor was directly connected to the ESP32, as its output signal remained within the acceptable voltage limits.

#### 2.2.2. High-Precision Sensor Configuration

**Figure 2:**
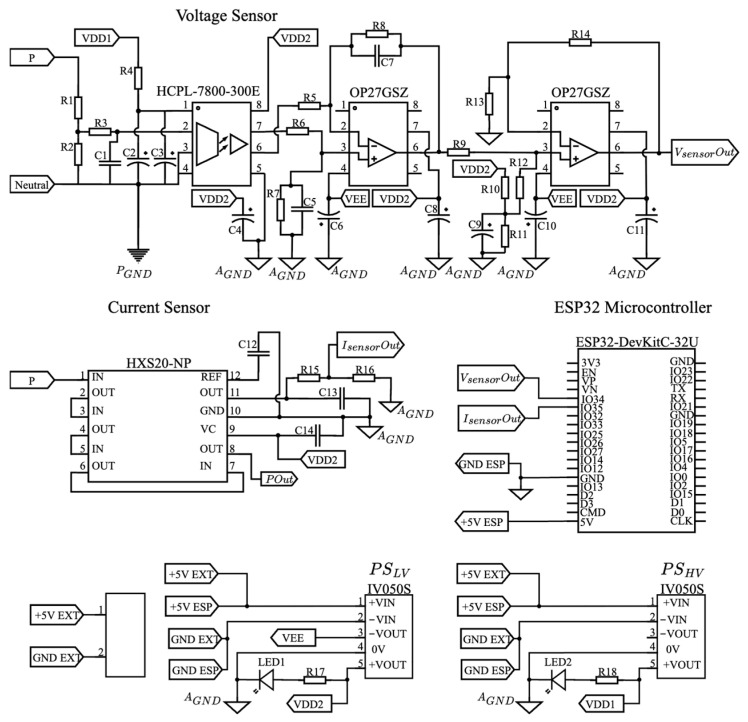
Monitoring board design with high-precision sensor.

The high-precision configuration required additional signal conditioning stages to adjust the output characteristics of the selected sensors. The schematic diagram of the monitoring board implementing this configuration is shown in Figure 2.

**Figure 3:**
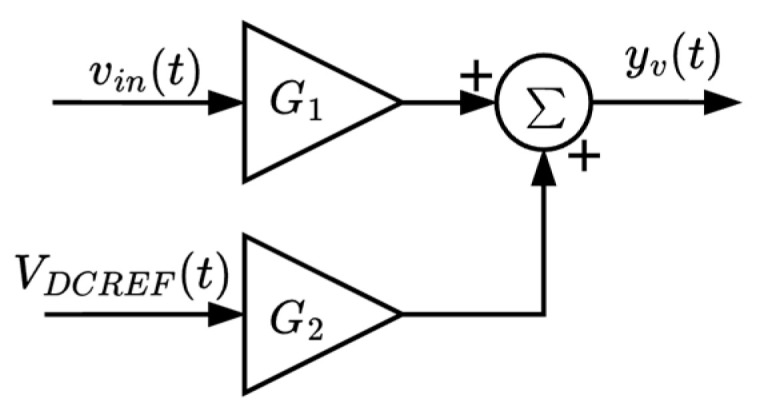
Block diagram of the voltage sensor circuit.

The voltage sensing stage employs the HCPL-7800, a high-precision isolation amplifier capable of providing up to 5 kV of galvanic isolation. The circuit can be configured for isolated voltage measurement in wide range of applications. The output signal depends on the selected passive components, which define the overall gain and offset. A block diagram of the voltage sensor circuit is presented in Figure 3.

The equation describing the output signal of the voltage sensor is given by:

*y_v_*(*t*) *=* [*v_in_*(*t*) G_1_] + [V_DCREF_(*t*) G_2_], (1)

where *y_v_*(*t*) is the output signal of the voltage sensor, *v_in_*(*t*) is the input signal, and V_DCREF_(t) represents the reference voltage added to the output to provide the required DC offset.

Regarding the current sensor, the HXS20-NP Hall-effect device is a high-precision sensor that provides galvanic isolation up to 3.5 kV. It can be configured for different nominal current ranges, including ±5 A, ±10 A, and ±20 A. The output signal is an analog voltage proportional to the measured current, which is subsequently conditioned to match the input range of the ESP32 microcontroller.

**Figure 4:**
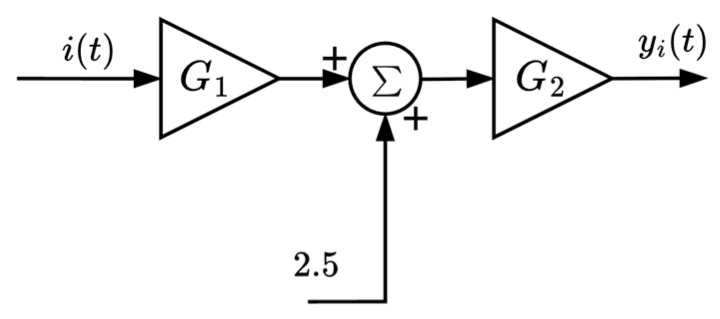
Block diagram of the current sensor circuit.

The block diagram of the current sensor circuit is shown in Figure 4.

The equation describing the output signal of the current sensor is given by Equation (2).

*y_i_*(*t*) *=* G_2_ (2.5 + G_1_ *i*(*t*)), (2)

where *y_i_*(*t*) is the output signal of the current sensor, and *i*(*t*) is the input current signal.

These design features reduce susceptibility to noise, thermal variation and nonlinearity, which explains why high-precision sensors provide more stable and traceable measurements than low-cost devices. With the monitoring board design and sensor output signals defined, the next step involves implementing the signal processing algorithm. This algorithm processes sensor data to calculate the RMS value, active power, apparent power and energy consumption, and subsequently transmits the information to a cloud platform for remote monitoring.

### 2.3. Signal-Processing Algorithm

A signal-processing algorithm was implemented on the ESP32 to acquire, filter, and process the analog signals from the sensors. Developed in C++ using an open-source embedded development framework for the ESP32, the algorithm enables real-time data acquisition, filtering, and cloud transmission, forming the core of the embedded monitoring platform. Key libraries such as WiFi.h, FirebaseESP32.h, and sensor-specific modules were integrated to streamline communication, data management, and cloud interaction. This modular structure facilitates scalability and reproducibility, allowing the framework to be adapted for different monitoring or control applications.

#### 2.3.1. RMS Calculation and Filtering

Sensor signals are sampled and digitally filtered to remove noise. A recursive averaging method is applied to compute the RMS value, reducing the processing demand on the microcontroller. This value is used to calculate apparent and active power, and consequently, energy consumption. The recursive RMS method is shown in Equation (3).

(3) $$\mathrm{RMS}[i]=\sqrt{{RMS}^{2}\left[i-1\right]+\frac{x^{2}\left[i\right]-x^{2}\left[i-N\right]}{N}}$$

#### 2.3.2. Data Transmission to the Database

Once processed, the data are transferred to a MySQL database and the Firebase platform to enable real-time monitoring and storage. The full implementation is available online at: https://github.com/JAJV02002/monitoreo_SolarVista_ProcesamientoSenales/blob/b60dd87991aa7c6c69aece46250c3a88b1a94610/src/main_Monitoreo_SolarVista.cpp (accessed on 12 November 2025).

### 2.4. Sensor Characterization

For the experimental characterization of the sensors, a measurement setup was implemented using a Keysight MSO3034 digital oscilloscope (300 MHz, 2.5 Gs/s) equipped with a TCP202A high-precision current probe. The reference voltage waveform was generated using a Chroma 61,703 programmable AC power source, operating in the range of 0–127 Vrms and 0–5 Arms. The load test bench consisted of a configurable resistor array rated at 10 Ω and up to 10 A (1 kW). For DC characterization, an ET5410 programmable electronic load was used.

Prior to data acquisition, all instruments were verified against their factory calibration certificates and validated using a Fluke 87 V true-RMS handheld meter as an external reference. No correction factors were required, since the deviation between the oscilloscope readings and the reference meter remained below 0.2% in the tested range. All equipment was powered 30 min before testing to ensure thermal stabilization, and the laboratory temperature was maintained at 25 ± 1 °C to prevent drifting.

To ensure repeatability and temporal stability of the measurements, the full test sequence was repeated over several non-consecutive days during a one-week period. For each operating point, 16 samples were acquired and averaged, resulting in a total of 160 measurements per sensor per variable (current and voltage). This procedure allowed the identification of potential day-to-day deviations and confirmed that measurements remained statistically consistent throughout the testing period.

For each sensor type, three identical units were tested in parallel under the same excitation conditions. These are labeled S_1_, S_2_, and S_3_ throughout the results to evaluate repeatability and unit-to-unit variability, which is particularly relevant for low-cost sensors due to their wider manufacturing tolerances.

The same reference instruments and procedures were used for both low-cost and high-precision sensor validation to guarantee methodological consistency.

Table 2 presents the validation of the current and voltage measurements obtained from the low-cost sensors (S_1_–S_3_) compared to the oscilloscope reference values (O).

The data show a clear difference between the readings obtained from the sensors and those recorded with the oscilloscope. The values reported in Table 2 correspond to the average of several repeated measurements, using the oscilloscope as the reference instrument. Repeating each test helped reduce random variations and allowed a fair comparison of both measurement methods.

All measuring instruments (the voltage probe, current probe, and oscilloscope) were calibrated before each test to guarantee reliable reference data. The use of repeated measurements, together with equipment calibration, ensured the consistency and traceability of the results.

**Figure 5:**
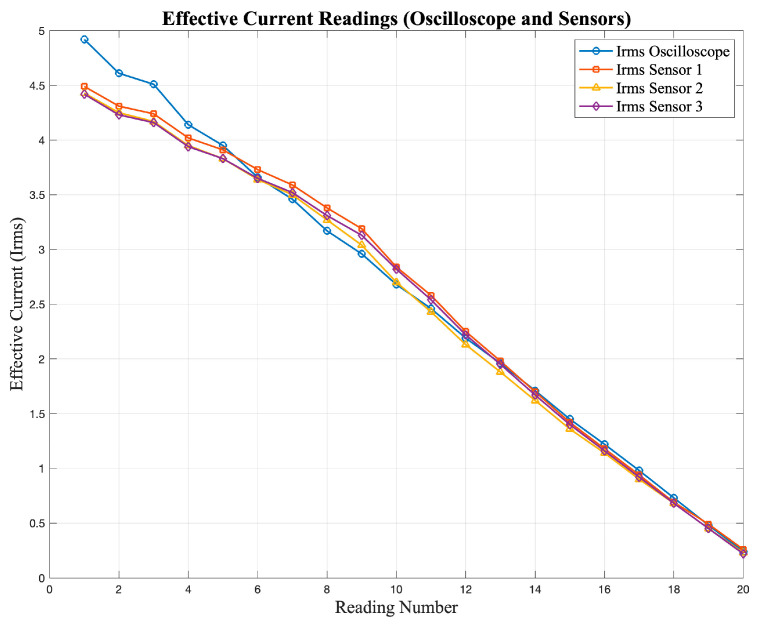
Current sensor response under different input signals obtained during the characterization tests.

**Figure 6:**
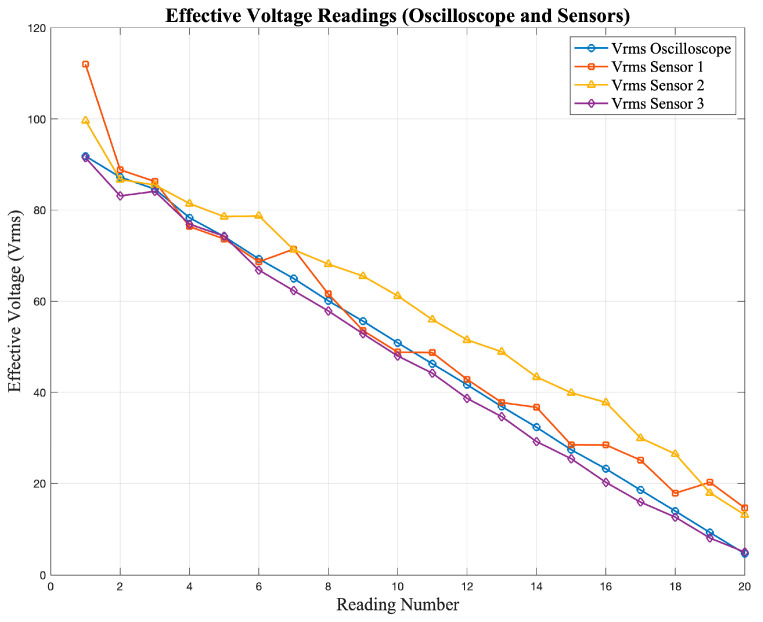
Voltage sensor response under different input signals obtained during the characterization tests.

The observed differences between the sensor outputs and the oscilloscope readings are mainly due to the limited accuracy and linearity of the low-cost sensors, which influence their response under different input conditions. Figure 5 and Figure 6 show the responses of the current and voltage sensors when subjected to different test signals, where deviations from the expected values can be clearly observed.

Figure 5 shows the current readings obtained from the sensors, while Figure 6 presents the corresponding voltage readings. In both cases, deviations from the expected values can be observed, especially at lower magnitudes.

To quantitatively evaluate the accuracy and reliability of the sensor readings, four complementary statistical metrics were employed: the percentage error, the MAPE, the MAE, the RMSE and the R^2^. These indicators provide information on both the magnitude of deviation and the degree of correlation between the sensor outputs and the reference values obtained from the oscilloscope [16].

**Figure 7:**
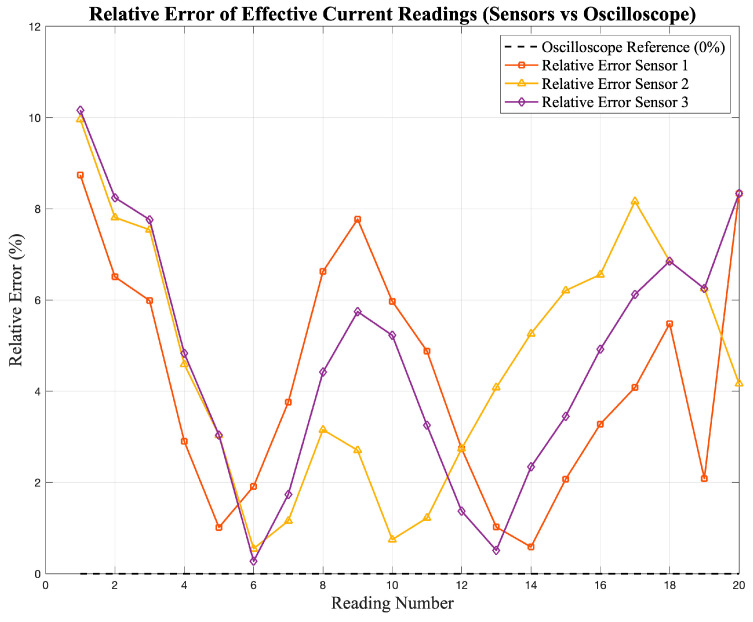
Relative error (%) of the effective current readings obtained from the low-cost sensors (S_1_–S_3_).

**Figure 8:**
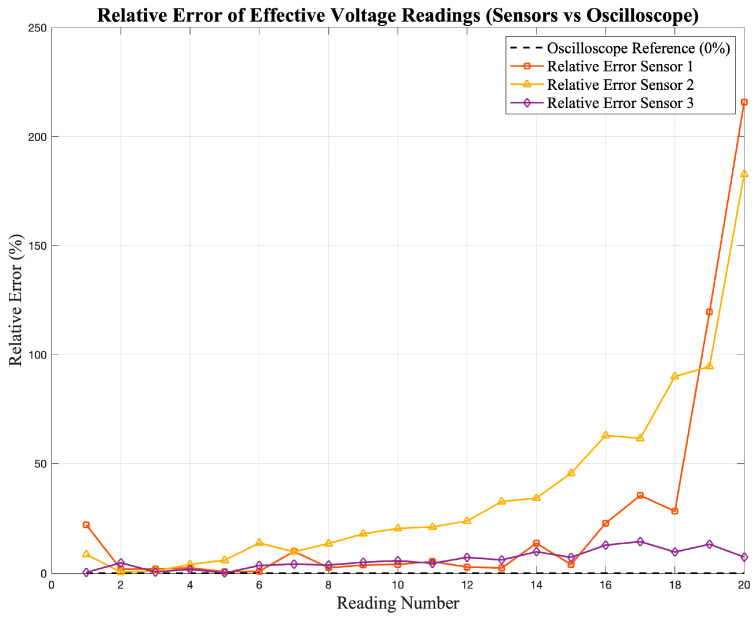
Relative error (%) of the effective voltage readings obtained from the low-cost sensors (S_1_–S_3_).

To estimate the level of deviation, the error was calculated for every data point using Equation (4).

(4) $$Error=\frac{\left|{RMS}_{o}-{RMS}_{sensor}\right|}{{RMS}_{o}}\times 100\%$$

where RMS_o_ represents the reference value measured by the oscilloscope and RMS_sensor_ is the corresponding reading obtained from the sensor. This metric provides a point-by-point estimation of the relative deviation between the sensor and the reference. The resulting error values are presented in Figure 7 and Figure 8, which show the deviation of the current and voltage sensor readings, respectively, with respect to the oscilloscope reference values.

In addition to the point-by-point values, a global performance indicator was computed by averaging the MAPE across all measurements, as presented in Equation (5).

(5) $$MAPE=\frac{1}{n}\sum_{i=1}^{n}\frac{\left|{RMS}_{o,i}-{RMS}_{sensor,i}\right|}{{RMS}_{o,i}}\times 100\%,$$

where *n* denotes the total number of measurements. The MAPE reflects the overall accuracy of the sensors over the entire set of experiments and is expressed as a percentage.

The MAE and RMSE quantify the absolute and quadratic deviations, respectively, between the reference and sensor readings, as defined in Equations (6) and (7).

(6) $$MAE=\frac{1}{n}\sum_{i=1}^{n}\left|{RMS}_{o,i}-{RMS}_{sensor,i}\right|$$

(7) $$RMSE=\sqrt{\frac{1}{n}\sum_{i=1}^{n}{\left({RMS}_{o,i}-{RMS}_{sensor,i}\right)}^{2}}$$

The MAE provides the average magnitude of error in the same units as the measurement (amperes or volts), while the RMSE penalizes larger deviations, offering a more sensitive indicator of precision.

Finally, the coefficient of determination (R^2^) was calculated to evaluate the degree of linear correlation between the sensor and oscilloscope readings, as shown in Equation (8)

(8) $$R^{2}=\frac{\sum_{i=1}^{n}{\left({RMS}_{o,i}-{RMS}_{sensor,i}\right)}^{2}}{\sum_{i=1}^{n}{\left({RMS}_{o,i}-\bar{{RMS}_{o}}\right)}^{2}},$$

where $\bar{{RMS}_{o}}$ denotes the mean of the reference values. An R^2^ value close to 1 indicates a strong correlation and high linearity between the sensor output and the oscilloscope reference.

Equations (5)–(8) constitute the metric framework used to evaluate the performance of the sensors. By applying the same indicators to all devices under identical test conditions, it was possible to quantify not only the absolute error of each sensor but also, its linearity and correlation with the reference.

The results, presented in Table 3 and Table 4, provide a quantitative basis for comparing low-cost and high-precision sensors and highlight the magnitude of the performance gap between both categories.

The statistical results for the low-cost sensors indicate moderate accuracy and linearity when compared with the oscilloscope reference. For current measurements, the correlation coefficients (R^2^) range between 0.87 and 0.90, with MAPE values below 5.2%, which suggests acceptable precision for non-critical or educational applications. However, in voltage measurements, the performance varies significantly among the sensors. Sensor S_3_ achieves the best results (R^2^ = 0.969, MAPE ≈ 3.0%), whereas S_1_ and S_2_ exhibit lower correlation (R^2^ < 0.75) and noticeably higher deviations, particularly at lower voltage levels.

This variability reflects the main limitation of low-cost devices: although they may provide reasonable accuracy under nominal conditions, their response becomes inconsistent when the signal amplitude changes, making them unsuitable for applications that require traceable, high-accuracy measurements.

#### 2.4.1. High-Precision Sensors Characterization

The high-precision sensors were evaluated under identical test conditions. The corresponding results are given in Table 5. The results indicate a notable improvement in both accuracy and linearity for the high-precision sensors compared with the low-cost models. The deviation from the expected values is minimal, confirming that the high-precision sensors deliver more reliable measurements and are better suited for monitoring applications in electrical power generation systems.

**Figure 9:**
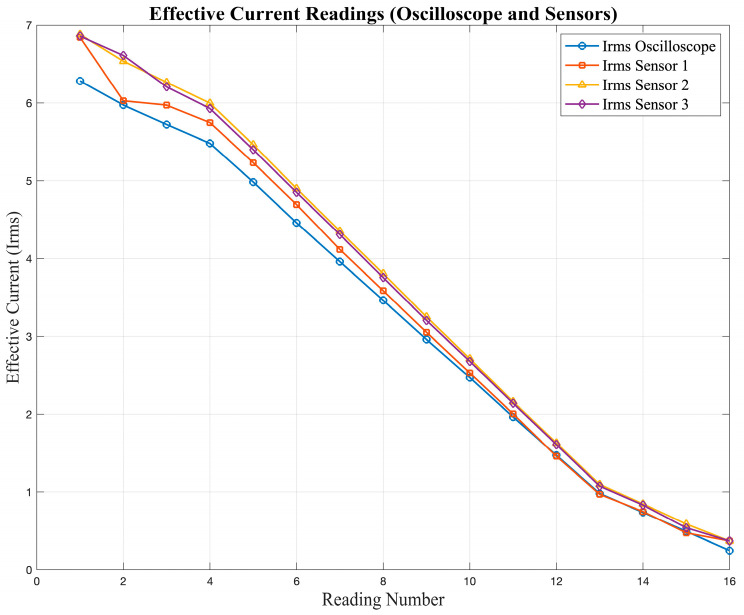
High-precision current sensor response under varying input signals.

**Figure 10:**
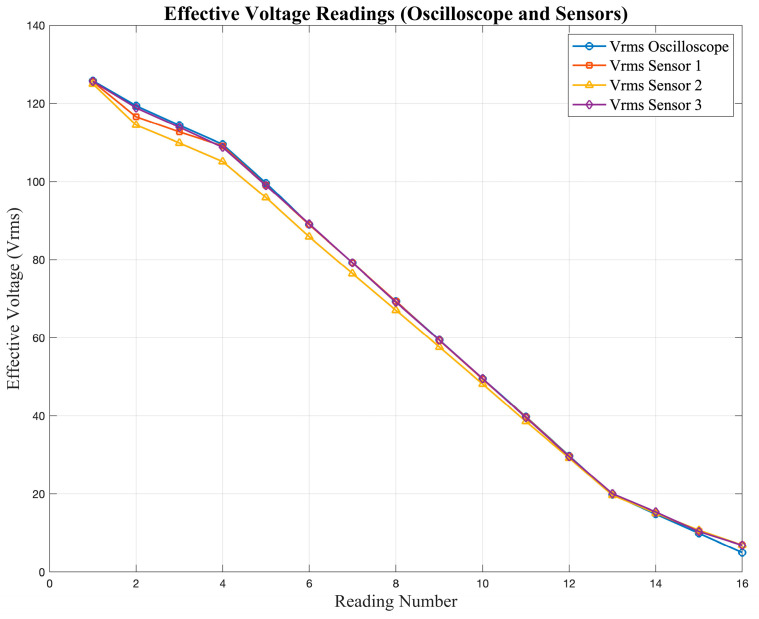
High-precision voltage sensor response under varying input signals.

The responses of the high-precision sensors to various input signals are presented in Figure 9 and Figure 10, illustrating their improved accuracy and linearity compared with the low-cost models.

As observed in Figure 9 and Figure 10, the high-precision sensors demonstrate a consistent and linear response throughout the tested range for both current and voltage signals. The deviation from the reference values is minimal across all measurements, confirming their high level of accuracy and repeatability. In contrast to the low-cost sensors, these devices maintain stable performance even at low signal amplitudes, where nonlinear behavior was previously observed. This behavior validates the superior sensitivity and calibration stability of the high-precision sensors, making them suitable for applications that demand accurate and continuous monitoring of electrical parameters in power generation systems.

**Figure 11:**
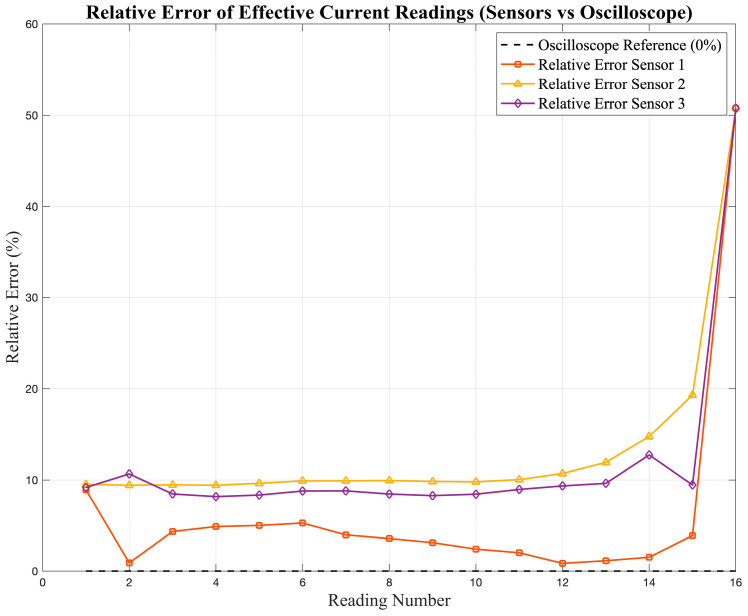
Relative error (%) of effective current readings obtained from the high-precision sensors (S_1_–S_3_) compared with the oscilloscope reference.

**Figure 12:**
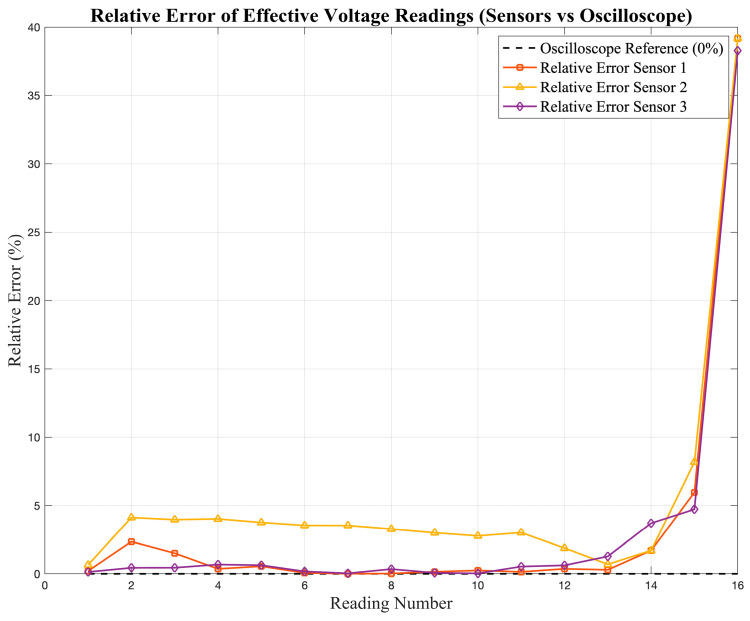
Relative error (%) of effective voltage readings obtained from the high-precision sensors (S_1_–S_3_) compared with the oscilloscope reference.

As with the low-cost sensors, the percentage error for each measurement was computed to quantify the deviation from the reference values. The results, shown in Figure 11 and Figure 12, demonstrate the improved accuracy and linearity of the high-precision sensors.

The statistical indicators (MAE, RMSE, R^2^, and MAPE) were used to evaluate the high-precision sensors for both current and voltage. The results, summarized in Table 6 and Table 7.

The high-precision current sensors demonstrate strong agreement with the oscilloscope reference, confirming their capability for accurate current measurement across the tested range. The correlation coefficients R^2^ are consistently high, with S_1_ achieving the best performance R^2^ = 0.972, followed by S_3_ (R^2^ = 0.918) and S_2_ (R^2^ = 0.908). The corresponding MAE and RMSE values remain below 0.41 A, indicating low dispersion and stable response. Furthermore, the MAPE values below 10% validate the overall reliability of these sensors for precise current monitoring. Among them, S_1_ exhibits the most balanced trade-off between accuracy and linearity, confirming its suitability for integration into high-performance measurement and control systems.

The results obtained for the high-precision voltage sensors demonstrate consistently high measurement accuracy and strong agreement with the oscilloscope reference. All sensors exhibit R^2^ ≥ 0.989, confirming a reliable linear response across the full measurement range. Sensor S_3_ achieved the best overall performance, with the lowest errors (MAE = 0.273 V, RMSE = 0.365 V, MAPE ≈ 0.30%) and R^2^ = 1.000, indicating an almost ideal correspondence between the sensor and the reference readings. Sensor S_1_ also showed measurement errors below 1%, while S_2_ presented slightly higher deviations (MAPE ≈ 3.2%) but maintained a high degree of linearity (R^2^ = 0.989).

Overall, these findings confirm that the high-precision voltage sensors provide stable and reliable measurements, making them suitable for monitoring applications that require accurate estimation of power and energy variables.

#### 2.4.2. Extended Error Analysis

In addition to the statistical indicators previously described, two complementary metrics were introduced to provide a more comprehensive assessment of the sensor performance: the Pearson correlation coefficient (*r*) and the Normalized Root Mean Square Error (nRMSE). These indicators enable a more detailed comparison between the sensor and reference readings, offering additional insight into linear consistency and relative accuracy.

The Pearson correlation coefficient (r) quantifies the strength and direction of the linear relationship between the sensor and oscilloscope readings, and it is expressed as:

(9) $$r=\frac{\sum_{i=1}^{n}\left({RMS}_{o,i}-\bar{{RMS}_{o}}\right)\left({RMS}_{o,i}-\bar{{RMS}_{sensor}}\right)}{\sqrt{\sum_{i=1}^{n}{\left({RMS}_{o,i}-\bar{{RMS}_{o}}\right)}^{2}\sum_{i=1}^{n}{\left({RMS}_{sensor,i}-\bar{{RMS}_{sensor}}\right)}^{2}}}$$

where $\bar{{RMS}_{o}}$ and $\bar{{RMS}_{sensor}}$ represent the mean values of the reference and sensor reading, respectively. A value of *r* close to 1 denotes a strong positive correlation, indicating that the sensor output closely follows the reference signal.

To compare performance across different measurement ranges, the Normalized Root Mean Square Error (nRMSE) was calculated as:

(10) $$nRMSE=\frac{RMSE}{max\left({RMS}_{o}\right)-min\left({RMS}_{o}\right)}\times 100\%$$

To establish a direct comparison between both sensor categories, Table 8 summarizes the average statistical indicators obtained for current and voltage measurements.

The high-precision sensors exhibit significantly lower normalized errors and higher correlation coefficients (R^2^ > 0.99, *r* ≈ 1) compared with the low-cost sensors (R^2^ < 0.90). These results confirm a consistent improvement in measurement fidelity and linearity, particularly in voltage readings, where the normalized RMSE was reduced by nearly 70%. This comparison highlights that while low-cost sensors provide acceptable accuracy for general monitoring, high-precision sensors remain essential for applications requiring traceable and high-resolution electrical measurements.

To complement the numerical results, a comparative assessment of the practical characteristics of low-cost and high-precision sensors was performed. Table 9 summarizes the main advantages and limitations observed in terms of accuracy, stability, conditioning requirements, and integration complexity. It is important to note that the performance gap between both sensor types is not only a result of cost, but also of fundamental design factors.

The performance of the high-precision sensors is largely a result of their internal design and signal-conditioning architecture. The HXS20-NP Hall-effect current sensor operates in a closed-loop configuration with internal compensation, which reduces offset drift, enhances linearity, and minimizes temperature-dependent error. Likewise, the amplifier-based voltage sensing stage uses precision instrumentation amplifiers with high common-mode rejection ratio (CMRR), low noise, and factory calibration, resulting in measurement accuracy below ±0.5%. In contrast, low-cost sensors such as SCT-013 and ZMPT101B rely on open-loop magnetic coupling or low-tolerance resistive dividers, which introduce higher nonlinearity, sensitivity to temperature variation, and unit-to-unit dispersion. These design differences justify the expected accuracy gap between both sensor categories and provide the basis for the experimental comparison.

## 3. Results

**Figure 13:**
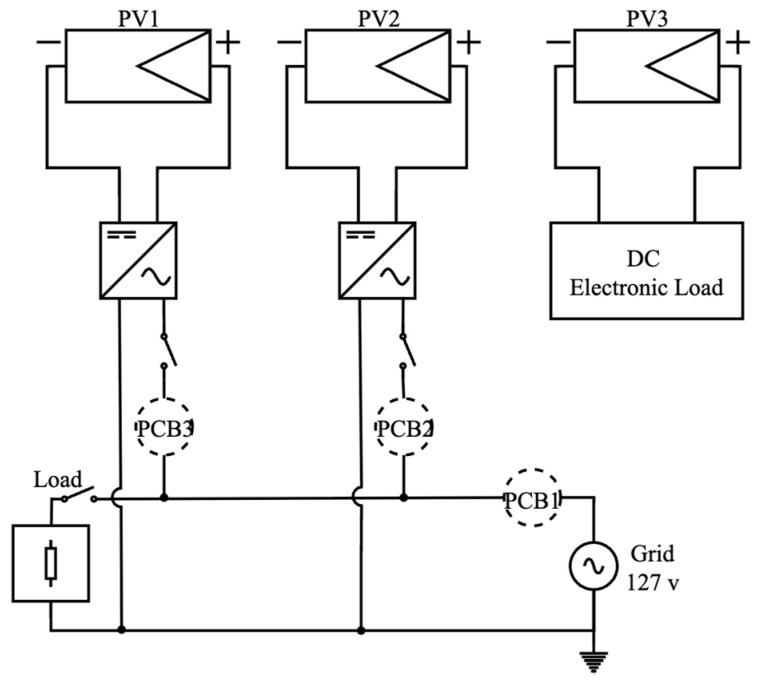
Wiring diagram of the experimental test bench, illustrating the interconnection between PV modules, monitoring boards, and measurement loads.

**Figure 14:**
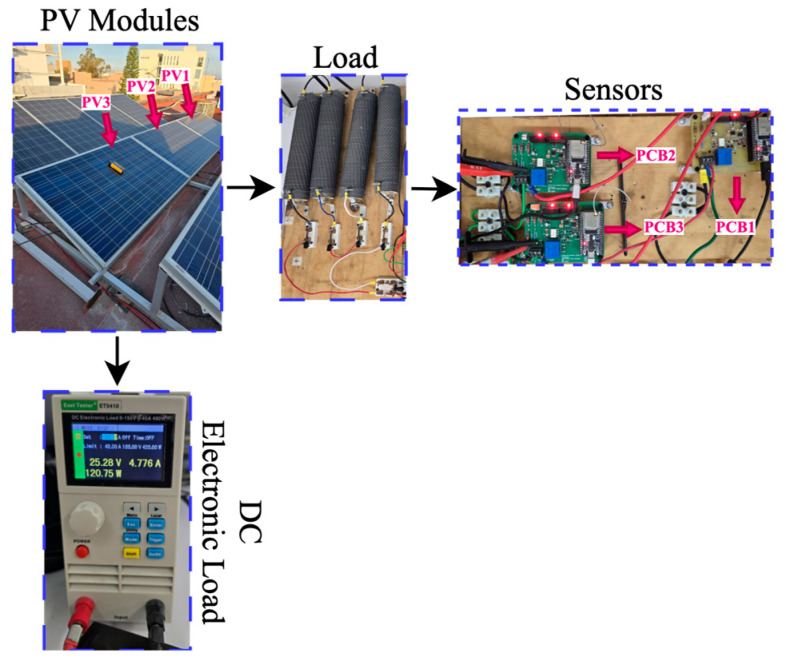
Field experimental platform used for sensor evaluation, consisting of three photovoltaic modules, a custom monitoring and acquisition board, high-precision reference sensors, and a programmable DC electronic load. PV1 and PV2 were connected to the grid-tied inverter, while PV3 was used for controlled characterization through the programmable load.

The field evaluation was conducted using the photovoltaic (PV) test platform depicted in Figure 13 and Figure 14, comprising three independent PV modules, a custom monitoring board integrating both low-cost and high-precision sensors, and a data acquisition unit for real-time logging. The system was operated under field conditions in San Luis Potosí, Mexico, with the objective of assessing its performance in response to varying irradiance profiles. This evaluation enabled the characterization of sensor behavior under diverse solar scenarios, including fluctuating, stable, and low-generation conditions, representative of cloudy, clear-sky, and rainy days.

Two modules were connected to the utility grid via their respective inverters and instrumented with high-precision voltage and current sensors serving as reference channels. The third module was interfaced with a programmable DC electronic load, enabling controlled characterization of the sensors under predefined electrical operating points. This configuration facilitated the simultaneous acquisition of electrical variables under both field operating conditions and controlled laboratory profiles, while ensuring time-synchronized measurements through a unified acquisition interface.

The purpose of the test was not to perform a comparative evaluation among the sensors, but rather to determine whether low-cost sensing devices can provide stable and reliable measurements under real photovoltaic monitoring conditions. High-precision channels were used exclusively as reference measurements and were not considered in the performance evaluation. All signals were sampled at 1 s intervals using a common clock source, ensuring that any observed deviations arose from sensor behavior rather than timing discrepancies.

**Figure 15:**
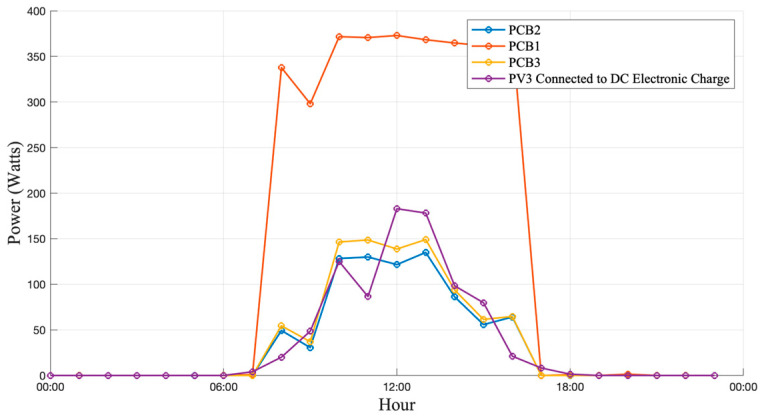
Generation and consumption curves on a cloudy day.

**Figure 16:**
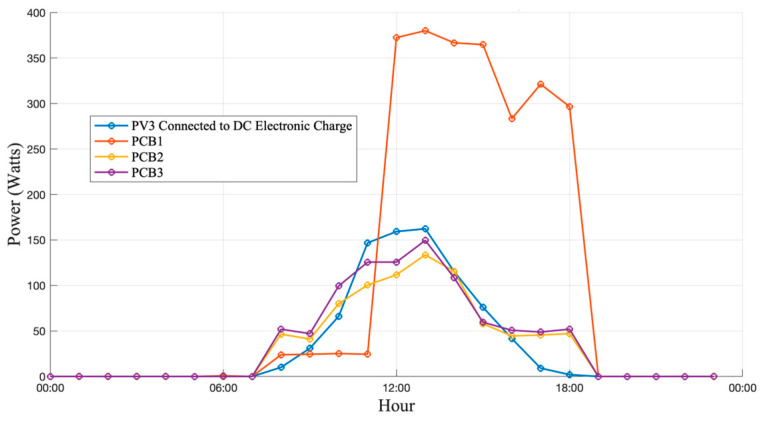
Generation and consumption curves on a sunny day.

**Figure 17:**
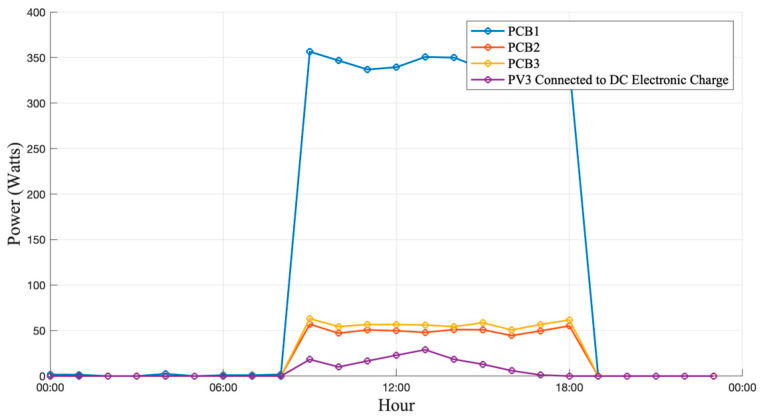
Generation and consumption curves on a rainy day.

Figure 15, Figure 16 and Figure 17 show the current and power curves obtained during the field tests under three different scenarios: cloudy, sunny, and rainy conditions, as described below.

### 3.1. Operation Under Cloudy Conditions

During cloudy-day operation, intermittent shading produced rapid fluctuations in the output power of the PV modules, resulting in a highly variable electrical profile throughout the day. As presented in Figure 15, the monitoring board successfully captured these variations in real time, demonstrating a stable response despite the abrupt irradiance transitions. The measured power level remained consistently below that of clear-sky conditions, with a reduction of approximately 12.5% in average generation. This scenario was particularly useful for evaluating the dynamic response of the sensors during fast irradiance changes. The high-precision sensors exhibited stable tracking of the reference measurements, whereas the low-cost devices showed noticeable deviations during transient conditions, especially during sudden irradiance drops.

### 3.2. Operation Under Sunny Day Conditions

Under clear-sky conditions, as illustrated in Figure 16, the PV generation curves exhibited stable behavior throughout the day, with a consistent correlation between irradiance and output power. The peak generation increased by approximately 14% compared with cloudy-day operation, confirming the system’s capability to track irradiance and power variations with high accuracy and minimal deviation. The results showed a strong correlation between the sensor readings and the reference laboratory instruments. Both current and voltage measurements achieved an accuracy exceeding 95%, confirming the reliability of the proposed sensing and acquisition system. The sensors were successfully integrated into the monitoring board, and the data acquisition platform consistently captured real-time measurements from the PV system under test.

### 3.3. Operation Under Rainy Conditions

Rainy-day measurements resulted in significantly reduced power generation due to the combined effect of low irradiance and increased diffuse radiation. As shown in Figure 17, the output power of the PV modules remained below 68% of the clear-sky level for most of the observation period. Despite the low signal amplitude, the monitoring system was able to capture the inverter output current and maintain stable acquisition performance.

This operating point is relevant for evaluating the lower detection limit of the sensors. The high-precision devices continued to provide consistent readings, while the low-cost sensors showed larger deviations, particularly at low current levels where the signal-to-noise ratio becomes critical.

Across the three operating scenarios, the high-precision sensors demonstrated consistent agreement with the laboratory reference instruments, confirming their stability under real field conditions. In contrast, the low-cost sensors exhibited greater sensitivity to irradiance transitions and low-power operation, especially during cloudy and rainy periods. These findings indicate that although low-cost devices are adequate for educational use, prototyping, or coarse monitoring, they require additional calibration or signal-conditioning stages before being considered for deployment-grade PV monitoring applications.

The field results validated that sensor selection plays a critical role in determining the reliability of measurement data in distributed energy systems. While low-cost sensors offer clear economic advantages, their performance degradation under rapidly changing irradiance and low-generation conditions limits their use in applications where high-resolution data or fault detection is required. Conversely, high-precision sensors showed stable performance in all environmental scenarios, reinforcing their suitability for long-term monitoring, diagnostics, and control in photovoltaic installations.

## 4. Discussion

The experimental results showed a clear difference in measurement consistency between the evaluated sensing devices, with high-precision sensors achieving correlation coefficients between 0.91 and 0.99 and MAPE values below 4%, whereas the single-ended low-cost sensors exhibited lower agreement, with R^2^ values dropping to 0.73 and MAPE exceeding 10% under rapidly changing irradiance. These findings are consistent with previous reports in PV and building-energy monitoring systems, where low-cost current and voltage sensors have shown performance degradation when exposed to real outdoor operating conditions [2,12]. Similar deviations have also been observed in IoT-based platforms for energy data acquisition in residential and industrial environments [3,7,10], confirming that measurement accuracy remains a recurrent limitation when sensors are deployed without calibration and noise-mitigation routines.

Beyond numerical deviation, the results also reveal a structural pattern in the error distribution: the inaccuracy in the single-ended devices increases with the magnitude of the measured signal, producing non-linear drift that propagates into derived variables such as power and energy yield. This behavior has also been noted in prior studies using uncalibrated transducers in field-deployed PV monitoring systems [1,8], where the absence of traceable validation leads to biased performance indices, particularly under partial shading or fast irradiance transitions. At scale, an average deviation of 8–12% may accumulate into significant distortion when extrapolated to monthly or annual yield analytics, especially in applications such as microgrids, rooftop aggregators, and fleet-level PV supervision [7,13].

A critical implication of this work concerns scientific reproducibility. Several recent studies employing low-cost sensors in embedded or cloud-connected PV monitoring architectures have reported system-level conclusions (e.g., on degradation, efficiency, or fault detection) without documenting calibration procedures, linearity tests, or uncertainty propagation [1,3,8]. As a result, their datasets may be affected by unquantified measurement bias, limiting comparability and reducing the validity of downstream inference, including machine-learning-based diagnostics, digital twins, or performance modeling. The present results reinforce that sensor selection is not only a budget decision, but a data-quality decision that directly affects the reliability of subsequent analytics and decision-making layers.

Although the evaluated sensors fall within a narrow price range (USD $5–$25), the practical cost of implementation differs substantially. As presented in Table 1, the SCT-013 current sensor offers inherent galvanic isolation and an easily interfaced output, whereas the ZMPT101B voltage transducer requires additional amplification, filtering, and ground-reference conditioning to maintain acceptable accuracy. Likewise, the isolated precision sensors such as the HCPL-7800 or HXS20-NP reduce the need for external calibration but introduce integration demands such as differential signal routing and auxiliary power supplies. These observations align with recent technical analyses of Hall-effect and isolation-based sensors, where implementation complexity, not component price, determines total system cost [15,16]. Thus, the decision between sensing alternatives must consider not only the unit price, but the combined cost of analog front-end design, signal conditioning, firmware compensation, and long-term stability testing.

Furthermore, sensors with single-ended analog outputs can be integrated into low-budget implementations, their use requires additional signal-processing stages to compensate for noise susceptibility, offset drift, and non-linearity factors that increase computational overhead and firmware complexity. In contrast, isolated differential-output sensing architectures preserve measurement stability at the hardware level, reducing the need for software-based corrections and enabling simpler, more scalable acquisition pipelines. Therefore, sensor selection should not be guided solely by component price, but by the trade-off between electrical integration requirements, processing complexity, and long-term data reliability.

From a system-design perspective, the findings indicate that selecting the least expensive component does not guarantee a cost-effective solution when scalability, deployment time, or maintainability are design constraints. In contrast, the monitoring board developed in this work demonstrated stable performance under irradiance variations exceeding ±30%, validating its suitability for long-term photovoltaic supervision and control.

Finally, the results open the way toward hybrid sensing architectures, where low-cost sensors may still be used, provided that they are complemented by calibration models or adaptive filtering strategies capable of compensating for inherent hardware limitations. This direction aligns with emerging trends in edge computing and machine-learning-assisted signal processing [7,10], and represents a valuable opportunity for future research in scalable and cost-effective photovoltaic monitoring systems.

## 5. Conclusions

Low-cost sensors such as the ZMPT101B and SCT-013 have gained popularity in embedded and IoT-based platforms for power estimation in renewable energy systems. However, their limited accuracy and lack of calibration are often underestimated, potentially introducing measurement errors that, when propagated across distributed energy systems, can lead to significant deviations and compromise overall performance.

The comparative analysis revealed clear trade-offs among performance, integration complexity, and processing requirements. Low-cost sensors offer ease of integration and affordability but exhibit reduced accuracy and linearity. In contrast, high-precision sensors such as the HCPL-7800, OP127GSZ, and HXS20-NP achieved errors below 1% and demonstrated consistent reliability under varying operating conditions, making them suitable for power quality diagnostics and advanced monitoring in renewable energy systems.

Overall, the findings underscore that sensor selection is a critical design decision in distributed energy systems, directly impacting measurement reliability, system efficiency, and long-term scalability.

## Figures and Tables

**Table 1:** Comparison of selected sensors.

Sensor	Type	Range	Current Type	Accuracy	Galvanic Isolation	Output Signal	Cost (USD)
ZMPT101B	Voltage	0–250 V	AC	1%	No	Analog voltage (Proportional to input)	$5
SCT-013	Current	0–20 A	AC	1%	Yes	Analog voltage (Proportional to current)	$10
HCPL-7800&OP127GSZ	Voltage	0–180 V	DC/AC	0.1%	Yes	Analog voltage (High linearity)	$18
HXS20-NP	Current	±20 A	DC/AC	0.01%	Yes	Analog voltage	$25

**Table 2:** Validation of current and voltage measurements: comparison between sensor outputs and oscilloscope reference readings.

#	Current (I_RMS_)				Voltage (V_RMS_)			
	O	S_1_	S_2_	S_3_	O	S_1_	S_2_	S_3_
1	4.92	4.49	4.43	4.42	91.85	112.00	99.67	91.49
2	4.61	4.31	4.25	4.23	87.22	88.84	86.71	83.11
3	4.51	4.24	4.17	4.16	84.62	86.27	85.50	84.11
4	4.14	4.02	3.95	3.94	78.31	76.38	81.43	76.89
5	3.95	3.91	3.83	3.83	74.17	73.66	78.55	74.28
6	3.66	3.73	3.64	3.65	69.27	68.65	78.73	66.83
7	3.46	3.59	3.50	3.52	64.99	71.41	71.30	62.31
8	3.17	3.38	3.27	3.31	60.08	61.59	68.14	57.86
9	2.96	3.19	3.04	3.13	55.62	53.56	65.52	52.85
10	2.68	2.84	2.70	2.82	50.87	48.82	61.17	47.98

**Table 3:** Statistical evaluation of the low-cost current sensors compared with the oscilloscope reference.

Sensor	MAE (A)	RMSE (A)	R^2^	MAPE (%)
S_1_	0.196	0.225	0.899	5.12
S_2_	0.176	0.235	0.890	4.12
S_3_	0.207	0.254	0.873	5.14

**Table 4:** Statistical evaluation of the low-cost voltage sensors compared with the oscilloscope reference.

Sensor	MAE (V)	RMSE (V)	R^2^	MAPE (%)
S_1_	3.852	6.839	0.732	4.99
S_2_	6.074	6.996	0.719	9.49
S_3_	1.951	2.311	0.969	2.97

**Table 5:** Validation of current and voltage measurements: comparison between high-precision sensor outputs and oscilloscope reference values.

#	Current (I_RMS_)				Voltage (V_RMS_)			
	O	S_1_	S_2_	S_3_	O	S_1_	S_2_	S_3_
1	6.28	6.84	6.88	6.86	125.80	125.54	124.99	125.63
2	5.97	6.03	6.54	6.61	119.40	116.59	114.47	118.88
3	5.48	5.75	6.00	5.93	109.50	109.10	105.09	108.76
4	4.98	5.23	5.46	5.40	99.63	99.08	95.88	99.01
5	4.46	4.69	4.90	4.85	89.00	88.94	85.85	89.15
6	3.96	4.12	4.35	4.31	79.22	79.22	76.42	79.19
7	3.46	3.59	3.81	3.76	69.33	69.32	67.06	69.09
8	2.96	3.05	3.25	3.21	59.41	59.32	57.62	59.37
9	2.47	2.53	2.71	2.68	49.53	49.41	48.15	49.54
10	1.96	2.00	2.16	2.14	39.75	39.69	38.55	39.54

**Table 6:** Statistical evaluation of the high-precision current sensors compared with the oscilloscope reference.

Sensor	MAE (A)	RMSE (A)	R^2^	MAPE (%)
S_1_	0.185	0.237	0.972	4.03
S_2_	0.408	0.408	0.908	9.78
S_3_	0.377	0.404	0.918	8.90

**Table 7:** Statistical evaluation of the high-precision voltage sensors compared with the oscilloscope reference.

Sensor	MAE (V)	RMSE (V)	R^2^	MAPE (%)
S_1_	0.436	0.920	0.999	0.410
S_2_	2.649	2.965	0.989	3.173
S_3_	0.273	0.365	1.000	0.304

**Table 8:** Statistical error metrics for low-cost and high-precision sensors relative to the oscilloscope reference.

Sensor	Measurement	MAE	RMSE	R^2^	MAPE (%)	*r*	nRMSE(%)
Low Cost	Current (A)	0.19	0.24	0.89	4.8	0.94	6.5
	Voltage (V)	3.96	5.38	0.81	5.8	0.90	7.2
High precision	Current (A)	0.32	0.36	0.93	7.6	0.97	4.2
	Voltage (V)	1.12	1.42	0.996	1.3	0.998	2.0

**Table 9:** Advantages and practical limitations of comparative sensors.

Aspect	Low-Cost Sensors	High-Precision Sensors
Accuracy and linearity	Moderate accuracy: deviations increase at low voltages and currents due to limited linear response.	High accuracy and linearity maintained across the full measurement range.
Response stability	Susceptible to drift caused by temperature variations and aging.	Excellent short- and long-term stability under environmental variations.
Signal Conditioning	Requires external amplification, filtering, and frequent recalibration.	Integrated conditioning, factory calibration, and temperature compensation.
Sampling and Resolution	Limited ADC resolution (10–12 bits), reducing sensitivity to small variations.	High-resolution ADCs (16–24 bits) enabling fine measurement detail.
Noise Immunity	More sensitive to electromagnetic interference and ground loops.	Shielded design and higher common-mode rejection ratio ensure cleaner signals.
Cost and Availability	Low cost and easy to implement, ideal for prototypes or educational systems.	Higher cost, intended for professional or industrial-grade monitoring.
Integration Complexity	Simple wiring and configuration, but prone to offset and calibration errors.	Requires careful configuration and communication setup (e.g., I^2^C, SPI, or Modbus) but ensures reliable long-term operation.

## Data Availability

The data and full implementation supporting the findings of this study are openly available on GitHub at Monitoreo SolarVista Signal Processing (Available online: https://github.com/JAJV02002/monitoreo_SolarVista_ProcesamientoSenales/blob/b60dd87991aa7c6c69aece46250c3a88b1a94610/src/main_Monitoreo_SolarVista.cpp (accessed on 12 November 2025)).
